# Authors’ Response to: “Health Opportunity Costs and Expert Elicitation: A Comment on Soares et al.” by Sampson, Firth, and Towse

**DOI:** 10.1177/0272989X20987222

**Published:** 2021-02-25

**Authors:** Marta O. Soares, Mark J. Sculpher, Karl Claxton

**Affiliations:** Centre for Health Economics, University of York, York, Yorkshire, UK; Centre for Health Economics, University of York, York, Yorkshire, UK; Centre for Health Economics, University of York, York, Yorkshire, UK; Department of Economics and Related Studies, University of York, York, Yorkshire, UK

We read with interest the commentary letter by Sampson et al.^1^ on our article, “Health Opportunity Costs: Assessing the Implications of Uncertainty Using Elicitation Methods with Experts.”^2^ Our article presents the design, implementation, and results of an elicitation exercise aiming to quantitatively gather the (uncertain) beliefs of individuals on a set of quantities. We use methods that our research team is well versed in.^3–7^ The quantities elicited relate to a set of key uncertainties identified in a previous piece of work—Claxton et al.^8^—which evaluated the available evidence on health opportunity costs (HOC) for the UK National Health Service (NHS), a quantity that is important for supporting policy decisions over investments using public health system funds. Claxton et al.^8^ identified evidence on the effects of changes in expenditure on the life year burden of disease but no evidence on the likely effects of expenditure on quality-adjusted life years (QALYs). Linking the effects of expenditure on mortality burden of disease to QALYs is hence the focus of our work.

The letter commends our study for its policy value and for its methodological quality and rigor but, at the same time, raises methodological concerns. The authors provide no references to support their view that these concerns minimize the policy relevance of our findings. In this letter, we respond to these concerns.

The first methodological concern regards the expertise of the individuals recruited, and we rebut this on 3 grounds. First, health care practitioners (the substantive experts recruited into this study) directly observe the health effects of the activities of the health system (linked to expenditure), and hence we argue they are best placed to evaluate the quantities of interest. Second, we followed best practice^9^ in including, in our sample of experts, representatives with specialism in all the different clinical areas of interest (e.g., cardiologists, who have the relevant experience in circulatory disease) and also individuals with expertise across clinical areas (e.g., general practitioners, radiologists, anesthetists, and public health specialists). The authors claim that those without a specialism have no expertise, but this is incorrect as these individuals complement those with specialisms by providing breadth across the different clinical areas, across the types of technologies and services covered by NHS expenditure, and across settings of care. Finally, the authors claim the policy experts possess no substantive expertise. This is true and has been made clear in our study: policy experts were convened separately and asked to elicit by reconciling the information elicited from clinical experts with their own judgments. The fact that some drew entirely on the judgments of substantive clinical experts is a result in itself and was expected, indicating that these individuals trust the judgments of individuals with substantive expertise. This does not, in any way, impair the validity of the elicitation exercise.

The second claim by the authors is that the elicited quantities are not meaningful. It should be noted that our article carefully lays out the definitions of the quantities elicited, and in supplementary material, we provide the extensive materials used for training the experts and the full questionnaire used in the elicitation. A number of arguments were presented supporting the authors’ claim. It is asserted that we have not used an existing framework for elicitation, such as the Sheffield Elicitation Framework (SHELF).^10^ A recent review identified that none of the existing frameworks for elicitation has been developed specifically for health care.^6^ The protocol we used for this exercise (which was defined a priori) was developed in accordance with a number of these frameworks, including the principles set out in SHELF.^10^ It was developed for the health care context and tailored to the specific needs of our exercise. The authors also claim that the level of heterogeneity within disease areas compromises the definition of the quantity of interest. We argue that heterogeneity is unavoidable, and clinicians are used to integrating it into their judgments. Moreover, many elicitations have been conducted over heterogeneous quantities, such as in attributing global foodborne disease to specific foods^11^ or in defining the relationship between future climate change and the increased risk of armed conflict.^12^ This is not to say that eliciting under heterogeneity is without its challenges, and we acknowledge, in the discussion of our article, that this may have resulted in the high level of observed within-expert uncertainty (see third claim below).

The authors of the commentary also claim that the assumption of conditional independence (used in defining the quantities for the elicitation) cannot be sustained and justify this by the possibility of spillover effects. This argument is, however, flawed. First, conditional independence is well established in the elicitation literature as an alternative to eliciting correlation.^9^ While the validity of conditional independence may be difficult to demonstrate, eliciting absolute quantities independently, as proposed by the authors of the letter, is certainly not a valid approach. Finally, in our study, spillover effects were explicitly excluded by requesting individuals only to consider expenditure and its health effects in the same disease area.

The third claim by the authors relates to the level of uncertainty in responses. In the discussion to our article, we acknowledge the challenges in eliciting policy-relevant but broad-ranging quantities, which are by definition uncertain. To support this claim, the authors question the validity of some of the responses. At the end of every section of the elicitation, participants were asked whether they were *confident* the answers they had given reflected their views and uncertainties. Response options were “yes,”“not sure,” and “no.” We examined the qualitative feedback from participants, and there was no suggestion that the answers lacked face validity (see section “Face Validity and Qualitative Feedback” of our article^2^ and supplemental material). In addition, results were largely unchanged when those who responded “no” or “not sure” were removed.

For these reasons, we dispute the substantive points raised by Sampson et al.^1^ The authors conclude by making the point that estimates are uncertain. There is uncertainty surrounding both the causal effects of changes in expenditure on mortality outcomes (which is reported in a sequence of publications^13–15^) and how these are likely to translate into QALY effects (reported in this article^2^). The use of expert elicitation, which elicits uncertainty as well as point estimates, is key to making the judgments required to support policy explicit.^16^ This allows for scrutiny, discussion, and accountability, which promote the advancement of methods and applications to inform policy choice and unavoidable decisions, such as, how much can a health care system afford to pay for the benefits offered by a new pharmaceutical? As with estimates of any policy relevant parameter, the question is, what does the balance of evidence suggests? We maintain our conclusion that the balance of evidence suggests that the health effects of changes in expenditure are, if anything, likely to be greater than suggested by the mounting empirical estimates (evolving from Claxton et al.^8^) of the effect of changes in health expenditure on mortality outcomes.^13–15^
